# Transportation and Centering Ability of Neoniti and ProTaper Instruments; A CBCT Assessment

**DOI:** 10.22037/iej.2017.09

**Published:** 2017

**Authors:** Zahrasadat Madani, Ali Soleymani, Tasnim Bagheri, Ehsan Moudi, Ali Bijani, Vahid Rakhshan

**Affiliations:** a *Department of Endodontics, Dental School, Babol University of Medical Sciences, Babol, Iran; *; b *Endodontist, Babol Iran, *; c *Department of Oral and Maxillofacial Radiology, Dental School, Babol university of Medical Sciences, Babol, Iran; *; d *Non-Communicable Pediatric Diseases Research Center, Babol University of Medical Sciences, Babol, Iran;*; e *Department of Dental Anatomy, Dental School, Azad University of Medical Sciences, Tehran, Iran*

**Keywords:** Centering Ability, Nickel Titanium Instruments, Root Canal Preparation, Root Canal Treatment, Transportation

## Abstract

**Introduction:**

Transportation is an important iatrogenic endodontic error which might cause failure. This study evaluated the canal transportation caused by Neoniti and ProTaper instruments, using cone-beam computed tomography (CBCT) cross sections.

**Methods and Materials:**

This *in vitro* experimental study was performed on 40 mesiobuccal roots of maxillary first molars. The teeth were scanned with CBCT. They were randomly divided into 2 groups (*n*=20) that were prepared using either Neoniti or ProTaper files. An endodontist prepared the canal according to the manufacturer’s guidelines. Prepared canals were re-scanned. The pre-instrumentation and post-instrumentation CBCT volumes were sectioned at 1 to 9-mm distances from the apex. The extent of canal dentine removal in mesial and distal directions were measured in each cross-section. Canal transportation and instrument centering ability were estimated based on the extents of root wall removal and were compared in both groups.

**Results:**

The groups were rather similar in terms of transportation and centering ability (*P*>0.05). However, canal preparation on mesial and distal walls was statistically significantly less in the Neoniti group, at most cross-sections. Transportation of both groups was not significantly different (*P*>0.05). Centering ability of both instruments was not significantly different (*P*>0.05).

**Conclusion:**

Neoniti and ProTaper instruments might have proper centering ability and minimum transportations. Both instruments might cause similar extents of transportation and centering abilities.

## Introduction

The purposes of mechanical cleaning of the root canal system are to eliminate microorganisms and residues of necrotic pulp from the canal walls and produce a conical shape to facilitate effective root canal irrigation and obturation of the canal in a three-dimensional space [1-6]. Repeated usage of endodontic instruments over root canal walls can lead to loss of the canal curvature near the apical foramen, and apical transportation due to the lack of instrument flexibility or weakness of instrumentation technique [7]. Transportation happens when the metal instrument with insufficient flexibility and an original shape of a straight bar fails to bend completely to remain within the center of a curved canal, and hence starts to create its own path within the root walls [6, 8-11]. Transportation displaces the form of foramen apical its natural position to a new iatrogenic position on the root walls which might lead to numerous difficulties and treatment failure [4, 8, 9, 12, 13]. It might eliminate the integrity of the root structure, reduce fracture resistance of the tooth, or perforate the root walls when the next larger files are used [4, 8, 9, 12, 13]. It also can cause accumulation of infected debris in the newly formed canal (because of problematic access to the root end), and disallow effective condensation of root filling materials into the newly formed canal [4, 8, 9, 12, 13].

Rotary endodontic systems are developed to reduce treatment time and the fatigue of both dentist and patient [14]. These systems use instruments made of the super elastic nickel titanium (NiTi) alloy, which is thought to bend well even in severely curved canals [6]. Therefore, they are thought to maintain the canal shape and cause minimum apical transportation [6, 15-18]. Because of their high flexibility, they can easily get access to the apical foramen within the first files and therefore, fewer files are needed to finish the canal and apical cleaning procedures [6, 19]. ProTaper (Dentsply Maillefer, Ballaigues, Switzerland) is amongst the pioneer systems that has numerous files naming SX (auxiliary shaping file, tip size 17) for shaping the coronal portion of the root canal, followed by S1 (tip size 20) in the coronal third and S2 (tip size 19) in the middle third; finishing file also consist of F1 (20/0.07), F2 (25/0.08) and F3 (30/0.09) and F4 (40/0.06) instruments.

Neoniti A1 (NEOLIX, Châtres-la-Forêt, France) is one of the single-file systems with full rotary motion. This system has continuous rotating movement and is made up of special alloy that permits the file flexibility. This system is produced with three different sizes (20/0.08, 25/0.08 and 40/0.08) that are recommended to be used with speed of 300 to 500 rpm and torque limit of 1.5 N/cm. According to its manufacturer, the wire-cut electric discharge machining mechanism allows a sharper edge as well as much greater flexibility [20]. This system is applied with a single-length preparation procedure that allows canal preparation at working length with a disposable single file. If proved efficient and safe, such simplified single-file instrumentation systems are favorable because of their ease of use. However, this system is not assessed yet except in a recent study [20], and this allegation should be proved by further investigation. 

This study was conducted aiming at assessing the extent of transportation and centering ability of the abovementioned files, using cone-beam computed tomography (CBCT).

## Materials and Methods

This *in vitro* study was performed on mesiobuccal (MB) roots of 40 human maxillary molars (extracted for treatment purposes only). As the inclusion criteria, all teeth had to have a root length of 19-22 mm as well as more than 25^°^ curvature greater than according to Schneider’s method [12, 20-22]. The teeth were stored in 2.5% sodium hypochlorite for 2-3 h and then stored in saline at 4^°^C, until the start of the procedure. 

After access cavity preparation, a #10 K-file (Mani, Tochigi, Japan) was placed inside the canal until emergence of its tip through the apical foramen. The working length was determined by subtracting 1 mm from the full length of the inserted K-file when the tip of the file was just visible from the apical foramen. Second MB canals were disregarded. Afterwards, the teeth were mounted in plaster blocks, up to their cementoenamel junctions, with standardized vertical and horizontal positions: They were positioned upright so that their occlusal surfaces were horizontal, and their buccal sides were parallel to the front side of the block. 

Before canal preparation, a CBCT image was taken from each tooth using a NewTom VG 9000 CBCT device (Quantitative Radiology SRL Co., Verona, Italy), at 110 kVp, 0.100 mm axial thickness, and 9.5 mA and 75×75×75 µm^3^ voxel size [20].

The teeth were assigned randomly to two groups (*n*=20). An endodontist carried out the root canal preparation procedure using 2.5% NaOCl irrigation establishment of apical patency with a #10 K-file. In the first group the preparation was performed using ProTaper instruments (Dentsply, Tulsa Dental, Tulsa, OK, USA), up to the F2. In the second group, the samples were prepared using Neoniti A1 (Neolix Creative Dental Instruments, Châtres-la-Forêt, France) instruments. In both groups the procedure was done using RC Prep lubricant (Premier Dental Products, Philadelphia, USA) and 2.5% NaOCl for irrigation. 

**Figure 1:**
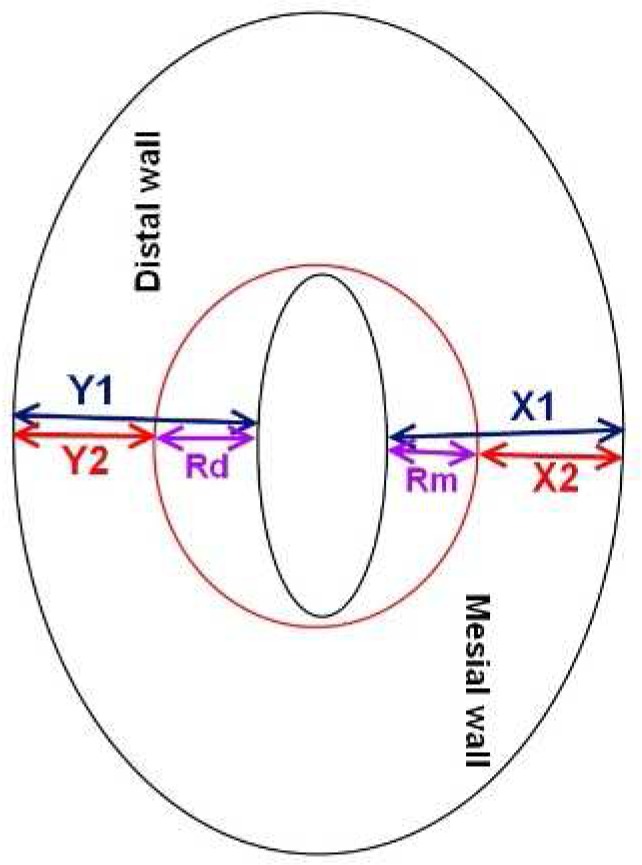
A schematic figure, showing the cross-section of non-instrumented canal (black small ovoid) and prepared canal (red circle). In this diagram, Y1 and X1 are wall widths before instrumentation, while Y2 and X2 are wall widths after instrumentation. Rd and Rm are the extents of wall removal from distal and mesial walls, respectively; Rd=Y1–Y2; Rm=X1–X2. Transportation=Rd–Rm; Centering ability=Rd/Rm

After preparation, the pre- and post-instrumentation CBCT images were taken with similar parameters. The images were evaluated using the Newtom scanner software (Newtom, Verona, Italy), for post-instrumentation changes: In each CBCT volume, first the root tip was identified and then the mesiobuccal root was sliced vertically. There were 9 cross-sections from the points 1, 2, 3, 4, 5, 6, 7, 8, and 9 mm coronally from the apex. Each of the sections were parallel to the horizontal plane on which the mounted tooth was positioned. The sections of pre-instrumentation and post-instrumentation volumes at each apical distance were inspected at 0.1 mm accuracy. Then the canal transportation at each of the 9 sections pertaining to each of the 40 teeth, was measured using the following formula: (Y1–Y2)–(X1–X2) [15, 22-24] (Figure 1), where Y is the shortest distance between the canal’s distal periphery and the root’s distal periphery [*i.e.*, the thickness of distal canal wall], and X is the shortest distance between the canal walls and root mesial peripheries (mesial canal wall thickness). The first and second values represent the pre-instrumentation and post-instrumentation measurements, respectively. The outcome of each parentheses block is the extent of canal wall removal in distal and mesial direction. Hence, a zero total outcome would mean the lack of canal transportation, while positive and negative outcomes would mean distal and mesial transportations, respectively. In addition, the formula (Y1–Y2)/(X1–X2) was used for the assessment of centering ability of the instrument, in which a ratio close or equal to 1.0 would suggest a high centering ability, at a given root section (Figure 1) [15, 22-24]. 

Canal curvatures were compared between two groups, using an independent-samples t-test. Descriptive statistics were calculated for mesial and distal canal wall removals, canal transportations, and centering abilities for each instrument at different points from the apex. In case the extent of mesial wall removal was zero, the centering ability would become infinity, disallowing any average calculations. Such cases were replaced with the ratio 4, as a ratio considerably greater than the maximum finite ratio in the sample (3.0). The data regarding wall removal, transportation, and centering ability were compared using the Mann-Whitney U test with SPSS software (Statistical Package for Social Science, SPSS, version 20.0, SPSS, Chicago, IL, USA). The extent of transportation of each instrument at each canal section was compared with the transportation value 0.0 mm, using the Wilcoxon paired ranks test. The extents of centering abilities were compared with the 1.0 ratio, using the Wilcoxon test. The level of significance was set at 0.05.

## Results

Two specimens in ProTaper group failed. The canal curvatures were 39.7±3.4 degrees in the ProTaper and 41.3±4.5 degrees in the Neoniti group. The difference between the two groups was not significant (*P*=0.228) according to the t-test. 

In both groups, the extent of canal wall removal increased constantly by moving away from the apex either on the mesial or on the distal walls (Table 1).

The extents of canal wall removal from each wall were significantly less in the Neoniti group, at most of the sections (*P*<0.05) (Table 1). However, the transportation and centering ability of both instruments were rather similar at all cross-sections (Tables 2 and 3). 

According to the Wilcoxon test, the transportation of each of the instruments at all sections was not significantly different from the 0.0 mm value (*P*>0.2) (Table 2). 

The centering ability of Neoniti was not significantly different from the 1.0 ratio at all canal sections (*P*>0.2) (Table 3). The centering ability of ProTaper was as well not significantly different from 1.0 (*P*>0.05). 

**Table 1:** The extent of canal removal on mesial (M) and distal (D) walls (mm), at various distances from the apex (M: mesial; D: distal; D1 to D9: sections 1 to 9 mm coronal to the apex; CI: confidence interval; Min: minimum; Max: maximum

Section	File	Mean	95% CI		Min	Max	*P-value*
M1	**ProTaper**	0.11 (0.02)	0.09	0.12	0.1	0.2	0.303
	**NeoNiTi**	0.09 (0.04)	0.07	0.10	0.0	0.1	
M2	**ProTaper**	0.12 (0.04)	0.10	0.14	0.1	0.2	-
	**NeoNiTi**	0.10 (0.00)	0.10	0.10	0.1	0.1	
M3	**ProTaper**	0.19 (0.06)	0.16	0.22	0.0	0.3	0.186
	**NeoNiTi**	0.17 (0.05)	0.14	0.19	0.1	0.2	
M4	**ProTaper**	0.22 (0.05)	0.19	0.25	0.1	0.3	0.264
	**NeoNiTi**	0.24 (0.24)	0.12	0.35	0.0	1.2	
M5	**ProTaper**	0.29 (0.07)	0.26	0.32	0.2	0.4	-
	**NeoNiTi**	0.24 (0.07)	0.21	0.27	0.1	0.3	
M6	**ProTaper**	0.34 (0.17)	0.26	0.43	0.0	0.9	0.051
	**NeoNiTi**	0.27 (0.09)	0.22	0.31	0.0	0.4	
M7	**ProTaper**	0.42 (0.06)	0.39	0.45	0.3	0.5	0.007
	**NeoNiTi**	0.35 (0.07)	0.31	0.38	0.2	0.4	
M8	**ProTaper**	0.49 (0.09)	0.44	0.53	0.3	0.7	0.000
	**NeoNiTi**	0.39 (0.06)	0.36	0.42	0.3	0.5	
M9	**ProTaper**	0.51 (0.15)	0.44	0.58	0.0	0.6	0.055
	**NeoNiTi**	0.48 (0.07)	0.45	0.51	0.3	0.6	
D1	**ProTaper**	0.11 (0.03)	0.10	0.13	0.1	0.2	0.303
	**NeoNiTi**	0.09 (0.03)	0.08	0.10	0.0	0.1	
D2	**ProTaper**	0.13 (0.05)	0.10	0.15	0.1	0.2	0.149
	**NeoNiTi**	0.10 (0.00)	0.10	0.10	0.1	0.1	
D3	**ProTaper**	0.22 (0.05)	0.19	0.24	0.1	0.3	0.033
	**NeoNiTi**	0.17 (0.06)	0.14	0.19	0.0	0.2	
D4	**ProTaper**	0.24 (0.09)	0.20	0.29	0.0	0.4	0.044
	**NeoNiTi**	0.20 (0.05)	0.17	0.22	0.1	0.3	
D5	**ProTaper**	0.31 (0.07)	0.28	0.34	0.2	0.4	0.030
	**NeoNiTi**	0.26 (0.06)	0.23	0.28	0.1	0.3	
D6	**ProTaper**	0.37 (0.08)	0.33	0.41	0.3	0.6	0.000
	**NeoNiTi**	0.29 (0.05)	0.26	0.31	0.2	0.4	
D7	**ProTaper**	0.43 (0.06)	0.40	0.46	0.3	0.5	0.001
	**NeoNiTi**	0.35 (0.06)	0.32	0.38	0.2	0.4	
D8	**ProTaper**	0.51 (0.07)	0.48	0.54	0.4	0.7	0.000
	**NeoNiTi**	0.40 (0.07)	0.37	0.43	0.2	0.6	
D9	**ProTaper**	0.55 (0.06)	0.52	0.58	0.4	0.6	0.001
	**NeoNiTi**	0.48 (0.04)	0.45	0.50	0.4	0.5	

**Table 2:** Canal transportation (mm) at various distances from the apex (M: mesial; D: distal; T: transportation in sections 1 to 9 mm coronal to the apex; CI: confidence interval; Min: minimum; Max: maximum

Section	File	Mean (SD)	95% CI		Min	Max	*P-value*
T1	**ProTaper**	0.01 (0.04)	-0.02	0.03	-0.1	0.1	0.965
	**NeoNiTi**	0.01 (0.04)	-0.01	0.02	-0.1	0.1	
T2	**ProTaper**	0.01(0.05)	-0.02	0.03	-0.1	0.1	0.478
	**NeoNiTi**	0.00 (0.00)		0.00	0.0	0.0	
T3	**ProTaper**	0.03(0.09)	-0.02	0.07	-0.1	0.3	0.346
	**NeoNiTi**	0.00(0.06)	-0.03	0.03	-0.1	0.1	
T4	**ProTaper**	0.02(0.11)	-0.03	0.08	-0.3	0.2	0.346
	**NeoNiTi**	-0.04(0.24)	-0.15	0.07	-1.0	0.2	
T5	**ProTaper**	0.02(0.05)	-0.01	0.05	-0.1	0.1	0.696
	**NeoNiTi**	0.02(0.08)	-0.02	0.05	-0.1	0.2	
T6	**ProTaper**	0.03(0.14)	-0.04	0.10	-0.3	0.4	0.806
	**NeoNiTi**	0.02(0.11)	-0.03	0.07	-0.2	0.3	
T7	**ProTaper**	0.02(0.05)	-0.01	0.04	-0.1	0.1	0.633
	**NeoNiTi**	0.01(0.06)	-0.02	0.03	-0.1	0.1	
T8	**ProTaper**	0.02(0.06)	-0.01	0.05	-0.1	0.2	0.633
	**NeoNiTi**	0.01(0.07)	-0.02	0.04	-0.1	0.2	
T9	**ProTaper**	0.04(0.15)	-0.04	0.11	-0.1	0.6	0.573
	**NeoNiTi**	-0.01(0.07)	-0.04	0.03	-0.2	0.1	

**Table 3:** Centering ability at various distances from the apex (M: mesial; D: distal; D1 to D9: sections 1 to 9 mm coronal to the apex; CI: confidence interval; Min: minimum; Max: maximum

Section	File	Mean (SD)	95% CI		Min	Max	*P-value*
C1	**ProTaper**	1.08 (0.35)	0.91	1.26	0.5	2.0	0.784
	**NeoNiTi**	1.40(1.14)	0.87	1.93	0.0	4.0	
C2	**ProTaper**	1.11(0.44)	0.89	1.33	0.5	2.0	0.784
	**NeoNiTi**	1.00(0.00)	1.00	1.00	1.0	1.0	
C3	**ProTaper**	1.28(0.84)	0.86	1.70	0.5	4.0	0.593
	**NeoNiTi**	1.08(0.54)	0.82	1.33	0.0	2.0	
C4	**ProTaper**	1.19(0.61)	0.89	1.50	0.0	3.0	0.478
	**NeoNiTi**	1.18(0.82)	0.80	1.57	0.2	4.0	
C5	**ProTaper**	1.10(0.23)	0.99	1.22	0.7	1.5	0.593
	**NeoNiTi**	1.19(0.66)	0.88	1.50	0.5	3.0	
C6	**ProTaper**	1.25(0.74)	0.88	1.62	0.7	4.0	0.851
	**NeoNiTi**	1.27(0.82)	0.88	1.65	0.5	4.0	
C7	**ProTaper**	1.05(0.15)	0.98	1.12	0.8	1.3	0.740
	**NeoNiTi**	1.04(0.22)	0.94	1.14	0.7	1.5	
C8	**ProTaper**	1.06(0.16)	0.98	1.14	0.8	1.5	0.654
	**NeoNiTi**	1.04(0.20)	0.94	1.13	0.7	1.5	
C9	**ProTaper**	1.19(0.71)	0.83	1.54	0.8	4.0	0.633
	**NeoNiTi**	1.01(0.15)	0.94	1.08	0.7	1.3	

## Discussion

This *in vitro* study compared the canal transportation and centering ability of Neniti and ProTaper instruments using CBCT. None of the tested systems showed significant differences regarding canal transportation or centering ability. The findings of the present study showed that while the extent of canal wall removal was slightly less in the Neoniti group (in both mesial and distal directions), the extents of transportation and centering ability were not significantly different between the instruments.. Because of the importance of these complications, various methods and materials are used to decrease this risk, although such complications still happen especially in highly curved and flat canals [4, 25-27]. Numerous risk factors are identified for this problem, including complex radicular anatomy, the lack of direct access, instrument design, incorrect sequences of the usage of different instruments, speed of instrument rotation, the use of inadequate irrigation and lubricant solutions. Of these risk factors, only two are intrinsic factors independent of the operator’s expertise and skill: internal canal anatomy and instrument design [1]. Of these two, the instrument design is the only one modifiable. 

There is another study on Neoniti that has evaluated the extent of transportation at distances 3, 4, and 5 mm coronally from the apex [20]. The extents of mesiodistal transportation reported by them were about 0.02 to 0.04 mm [20], which were comparable to findings of the present study. These results are comparable with other NiTi instruments showing high bending capacity and centering ability during rotation and thus following the natural shape of curved canals with minimum or no transportations [6, 19, 28, 29]. In comparison with other instruments like ProFile or HeroShaper, ProTaper has shown the least apical transportation [5, 6, 8, 30].

Most rotary NiTi files are used with the crown-down technique, where larger files are initially used to widen the coronal parts of the canal, while smaller files are used subsequently to reach the apical area. A tempting simplified instrument design has enabled “single-length technique” which is adopted by ProTaper, OneShape and Neoniti instruments. The sequentially used files are introduced into the canal at the full working length to prepare the whole canal [4, 29]. This mechanism alongside the improved bending ability and cutting potency might reduce the transportation by maintaining the canal’s original pathway while removing debris better than other NiTi instruments even when used by inexperienced operators [4, 26, 27]. According to a research, over instrumentation of F3 files of ProTaper system might cause damage to the root canal and hence might not be that simple for inexperienced practitioners [31]. Moreover, a recent study evaluated the use of F1, F2, and F3 of ProTaper instruments by dental students in molar teeth and found proper results, if the instrumentation was limited to using F1 and F2 and not F3 [30]. Using F3 files is not always practical for curved MB1 canals of maxillary molars. For the very same reason, in the present study F1 and F2 files were used. However, the difference in study design between the two studies (root canal preparation being performed by students versus an endodontist) reduce their comparability [32, 33]. Moreover, F3 (30/0.09) is considerably greater in size than Neoniti (25/0.08), and its use could render the comparison of two systems.

Peters *et al*. [34] found ProTaper useful especially in flat canals compared to wide and immature canals. Iqbal *et al.* [35] showed that ProTaper has transportation extents similar to Profile; according to them, transportation did not occur in curved canals [35]. However, Gergi *et al.* [15] evaluated transportation and centering ability of Pathfinder-ProTaper at apical, middle, and cervical sections and reported about 0.7 to 0.8 mm transportation, which was much greater than the values observed in this study. Silva *et al.* [36] compared ProTaper Next with Twisted File Adaptive at three cross-section (3, 5 and 7 mm from the apex) and observed small and similar extents of transportation and centering ability for both systems. Findings of Tambe *et al.* [37] in terms of centering ability and transportation caused by ProTaper were as well consistent with our results.

This research was constrained by some limitations. It was better to compare the results with a conventional file. However, transportation has no agreed-on gold standard [6]. Since the lower transportation of NiTi files compared to that of stainless steel hand instruments is already established [38], we focused on NiTi engine-driven instruments only. Another limitation was that despite our attempt to standardize the groups using the exclusion/inclusion criteria, extracted teeth cannot be completely standardized in terms of canal shapes and sizes [39, 40]. Studies on the geometry of canal affected by instrumentation need to be standardized with respect to multiple factors (such as canal shape and size, Knoop hardness, proper superimposition of before- and after-instrumentation images, and apical diameter) [39-42]. Different methods with their own limitations have been used to evaluate the canal modification caused by endodontic files [6, 23, 43]. These include cross-sectioning [6, 44], longitudinal sectioning [6], radiographic assessments [6, 45], computed tomography [6], or custom-built resin block simulations [39, 46, 47]. Some of these methods (like sectioning or SEM assessment) are aggressive to the specimen and hence do not allow the overlapping of the before-after sections. Some others are difficult to overlap (like 2D radiographs). The transparent resin block method might be one of the most reproducible and superimposable methods [39, 46, 47]. But the extent of transportation is estimated in 2D, and the resin hardness might be low; although they are still acceptable replacements for teeth, especially when made from high-hardness resin; on the other hand, they have many disadvantage such as easy removal of resin blocks which can disallow further file progression [39, 46, 48, 49]. 

Although the gold standard for examination of the centering ability of endodontic files is micro-CT, CBCT offers many advantages as well [6, 7, 20, 22, 50]. CBCT on the other hand can provide non-aggressive 3D information from before and after treatment, that can also be easily superimposed in 3D [6, 7, 20, 22, 50]. At lower fields of view, CBCT provides accurate 3D diagnostic information at low radiation doses [6, 7, 20, 22, 50]. In this study, a high resolution was selected that was one of the most accurate ones in the literature [20, 22]. As an advantage, this study was conducted on natural teeth, therefore its results could be better generalizable to the clinical practice. Finally, all previous studies had only compared different systems with each other.

## Conclusion

Within the limitations of this *in vitro* study, it was concluded that Neoniti might possess a proper centering potential and perhaps its usage in MB1 canals of maxillary molars might cause minimum transportation up to 9 mm above the apex. The use of ProTaper file might be safe as well in the MB1 canals of maxillary molars in terms of proper centering ability and transportation. Performances of these two systems were similar in terms of transportation and centering ability.
